# Targeting Fatty Acid-Binding Protein 4 Improves Pathologic Features of Aortic Stenosis

**DOI:** 10.3390/ijms23158439

**Published:** 2022-07-29

**Authors:** Mattie Garaikoetxea, Ernesto Martín-Núñez, Adela Navarro, Lara Matilla, Amaya Fernández-Celis, Vanessa Arrieta, Amaia García-Peña, Alicia Gainza, Virginia Álvarez, Rafael Sádaba, Eva Jover, Natalia López-Andrés

**Affiliations:** Cardiovascular Translational Research, Navarrabiomed, Hospital Universitario de Navarra (HUN), Universidad Pública de Navarra (UPNA), IdISNA, 31008 Pamplona, Spain; mattie.garaikoetxea.zubillaga@navarra.es (M.G.); emarnu87@gmail.com (E.M.-N.); adela.navarro.echeverria@navarra.es (A.N.); lara.matilla.cuenca@navarra.es (L.M.); amaya.fernandez.decelis@navarra.es (A.F.-C.); varriet8@hotmail.com (V.A.); amaiagpu@hotmail.com (A.G.-P.); alicia.gainza.calleja@navarra.es (A.G.); virginia.alvarez.asiain@navarra.es (V.Á.); jr.sadaba.sagredo@navarra.es (R.S.)

**Keywords:** FABP4, aortic stenosis, sexual dimorphism, inflammation, apoptosis, calcification, valvular interstitial cell

## Abstract

Aortic stenosis (AS) is a fibrocalcific disease of the aortic valves (AVs). Sex-differences in AS pathophysiology have recently been described. High levels of fatty acid-binding protein 4 (FAPB4) in atherosclerotic plaques have been associated with increased local inflammation, endothelial dysfunction, and plaque vulnerability. FABP4 pharmacological blockade has been shown to be effective for the treatment of atherosclerosis by modulating metabolic and inflammatory pathways. We aimed to analyze the sex-specific expression of FABP4 in AS and its potential role as a therapeutic target. A total of 226 patients (61.5% men) with severe AS undergoing surgical AV replacement were recruited. The FABP4 levels were increased in the AVs of AS patients compared to the control subjects, showing greater expression in the fibrocalcific regions. Male AVs exhibited higher levels of FABP4 compared to females, correlating with markers of inflammation (IL-6, Rantes), apoptosis (Bax, caspase-3, Bcl-2), and calcification (IL-8, BMP-2 and BMP-4). VICs derived from AS patients showed the basal expression of FABP4 in vitro. Osteogenic media induced upregulation of intracellular and secreted FABP4 levels in male VICs after 7 days, along with increased levels of inflammatory, pro-apoptotic, and osteogenic markers. Treatment with BMS309403, a specific inhibitor of FABP4, prevented from all of these changes. Thus, we propose FABP4 as a new sex-specific pharmacological therapeutic target in AS.

## 1. Introduction

Aortic stenosis (AS) is the leading cause of valvular heart disease (47%), with a prevalence of 2–7% in individuals >65 years and a 2-year mortality that increases up to 50% in symptomatic patients [1,2,3]. Aging population projections estimate a continued increase in the number of people >75 years in the coming decades, which will increase the prevalence of AS to more than double [4]. The lack of pharmacological strategies restricts the management of severe AS to surgical aortic valve replacement and transcatheter aortic valve implantation [5]. Several pathogenetic mechanisms and risk factors promote early-stage damage in the aortic valve (AV) structure. A complex sequence of events leads resident valve interstitial cells (VICs) to actively respond to an altered microenvironment by differentiating into myofibroblast-like cells or osteogenic cells. Altered extracellular matrix (ECM) remodeling and chronic inflammation induce VICs apoptosis, which, along with osteoblastic differentiation, leads to mineralization of the leaflets [6]. Men have a higher risk of developing AS than women. Although clear sex-specific differences in the clinical presentation or patient management have been evidenced, the underlying sex-dependent mechanisms driving the pathogenesis of AS have not yet been explored in depth [7,8]. Recent histological characterization of AVs and in vitro studies in VICs have shown that men present increased inflammatory, apoptotic, and calcification processes than women as well as a decreased ECM remodeling [9,10].

Fatty acid-binding protein (FABP) 4 is a long chain fatty acid chaperone that plays a key role in intracellular lipid transport and homeostasis, regulating lipolysis in adipose tissue [11]. It is mainly expressed and secreted from adipocytes, but it can also be found in macrophages and endothelial cells. Such increased expression leads to cholesterol ester accumulation, foam cell formation, and/or endothelial dysfunction [11]. The secreted form might act as an adipokine, targeting multiple organs and cell types [12]. FABP4 contributes to the development of a variety of pathological conditions associated with the metabolic syndrome cluster such as insulin resistance, obesity, hypertension, heart failure, and atherosclerosis [11]. Of special interest, ectopic expression of FABP4 in atherosclerotic plaques is due to its excessive production by macrophages that infiltrates within the lesion, being associated with greater local inflammation or plaque vulnerability [13]. In vitro experiments have shown that the local secretion of FABP4 by epicardial/perivascular fat and macrophages within the atherosclerotic lesion can have paracrine/autocrine effects, upregulating vascular inflammation, or impairing endothelial function, which contributes to the progression of atherosclerotic damage [14] FABP4 has also been implicated in the modulation of apoptosis in different cell types through the nuclear factor-κB (NF-κB) pathway [14,15,16]. Pharmacological blockade of FABP4 with small molecule inhibitors such as BMS309403 have shown to be an effective therapeutic strategy against insulin resistance, diabetes mellitus, and atherosclerosis by suppressing the associated inflammatory and apoptotic processes [17]. Sex-differences in FABP4 expression is still a matter of debate. Women with atrial fibrillation [18] or coronary atherosclerosis [19] present higher plasma FABP4 levels, whereas women with stable angina and chronic kidney disease exhibit reduced plasma FABP4 [20].

The possible involvement of FABP4 in the development of AS has not yet been addressed. Considering the similar features between atherosclerosis and AS, we propose that FABP4 may play an important role in the pathophysiology of AS and, consequently, may be postulated as a novel therapeutic target. To this end, we investigated the expression of FABP4 in the AVs and VICs of patients with AS and its relationship with different markers of inflammation, apoptosis, and calcification as well as the effects of its inhibition. We also explored the possible sex differences in the FABP4 expression in AVs from AS patients.

## 2. Results

### 2.1. Clinical Data in AS Patients

A total number of 226 AS patients (mean age 71.21 years, 61.50% men) were recruited. Men were significantly younger than women and exhibited higher height, weight, and body surface, lower cholesterol, HDL, and LDL levels, as previously reported [10]. Regarding control subjects, 16 cadaveric individuals were included (mean age 76 ± 10, 55% male). Their clinical data are similar to other studies from our group that have been previously published [21].

### 2.2. FABP4 Is Expressed in the Human Aortic Valve

**Figure 1:**
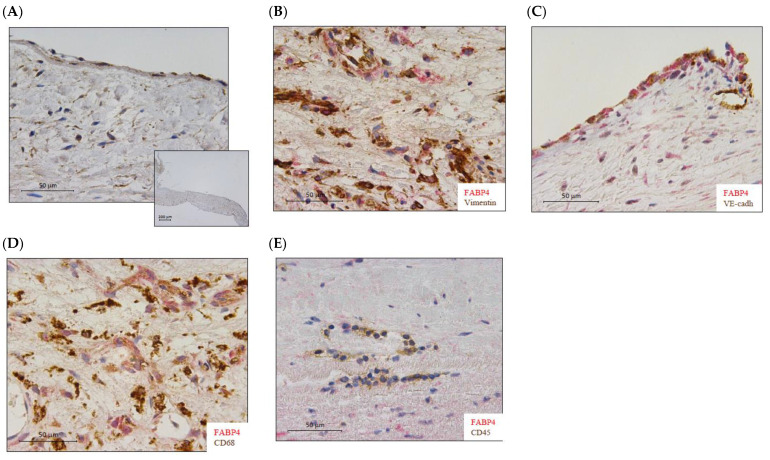
The expression of FABP4 in human aortic valves. (**A**) Representative microphotographs of AV sections immunostained for FABP4 in the control AVs. (**B**) Co-localization of FABP4 (in red) with Vimentin, (**C**) VE-Cadherin, and (**D**) CD68 but not with CD45 (**E**) positive cells (in brown) in AVs from AS patients. AV: aortic valve; AS: aortic stenosis; FABP4: fatty acid-binding protein 4; VE-cadherin, vascular endothelial cadherin; CD, cluster differentiation.

FABP4 immunostained AV sections revealed its presence in human AVs from healthy subjects (Figure 1A). Co-localization with vimentin, VE-cadherin, and CD68 (Figure 1B–D) positive cells revealed that FABP4 was expressed in the VICs, VECs, and macrophages in AVs from AS patients. However, FABP4 did not co-localize with CD45 positive cells, indicating that leukocytes are not a source of this protein in AVs from AS patients (Figure 1E).

### 2.3. FABP4 Expression Is Increased in AS Valves with Higher Levels in Men

**Figure 2:**
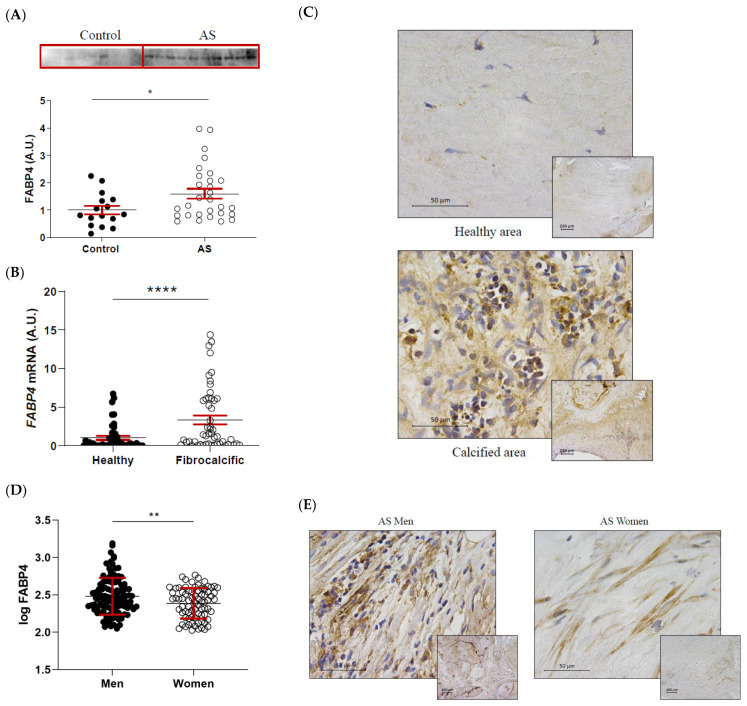
The differential expression of FABP4 in AS valves according to tissue damage and sex. (**A**) The protein expression of FABP4 in tissue homogenates from AVs of the control and AS patients. (**B**) The mRNA expression levels of FABP4 in the AVs of AS patients separated by their macroscopic healthy or fibrocalcific status. (**C**) Representative microphotographs of healthy and calcified AV sections of AS patients immunostained for FABP4. (**D**) Protein expression levels of FABP4 in tissue homogenates from the AVs of male and female AS patients. (**E**) Representative microphotographs of AV sections from men and women with AS immunostained for FABP4. Dot plots represent the mean and standard error of the mean (SEM) of each group of subjects (Control AV N = 16 vs. AS valves N = 29; Healthy N = 54 vs. Fibrocalcific N = 50; Men N = 109 vs. Women N = 77). Protein levels were measured by Western blot (normalized to stain free protein, A.U.: arbitrary units) or ELISA. The mRNA expression levels (A.U.) were normalized to the *18S*, *HPRT*, *ACTB*, and *GADPH* levels and healthy group was used as the calibrator. AV: aortic valve; AS: aortic stenosis; AU: arbitrary units; FABP4: fatty acid-binding protein 4. * *p* < 0.05, ** *p* < 0.01, **** *p* < 0.0001.

FABP4 protein expression was significantly higher in AS valves compared to healthy control ones (1 ± 0.15 vs. 1.6 ± 0.18-fold-change; *p* = 0.0364) (Figure 2A). Within the AS valves, *FABP4* mRNA expression was increased in areas of fibrocalcific tissue compared to healthy regions (1 ± 0.25 vs. 3.34 ± 0.58-fold-change; *p* < 0.0001) (Figure 2B). This result was confirmed by immunohistochemical analysis, showing greater expression of FABP4 in inflammatory foci near the calcification zone (Figure 2C). Considering the sex-differences in molecular hallmarks of AS, we investigated the potential sexual dimorphism in FABP4 expression in AS valves from our cohort. Protein levels of FABP4 were significantly higher in AVs from men than women (2.48 ± 0.02 vs. 2.39 ± 0.02 pg/mL; *p* = 0.0065) (Figure 2D). Accordingly, histological analyses revealed higher FABP4 immunostaining in AVs from men compared to women (Figure 2E).

### 2.4. FABP4 Expression Correlates with Inflammatory, Apoptotic and Osteogenic Markers in AS

**Figure 3:**
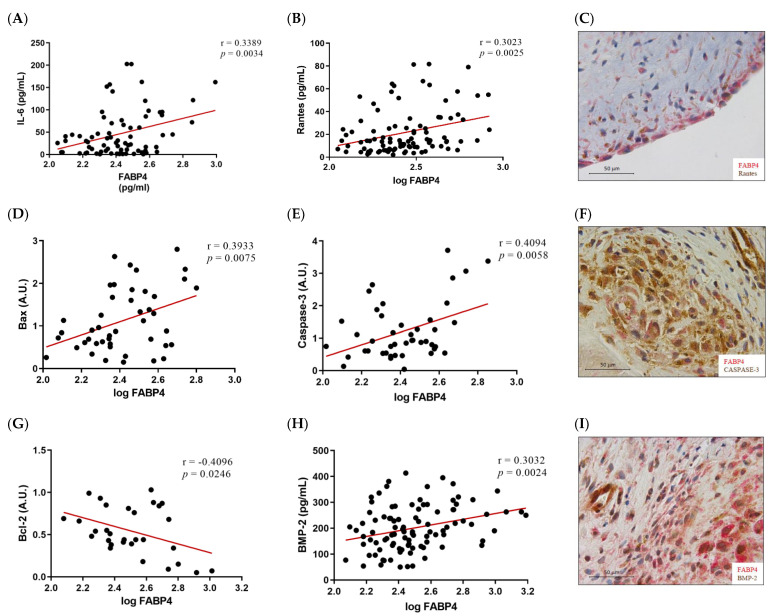

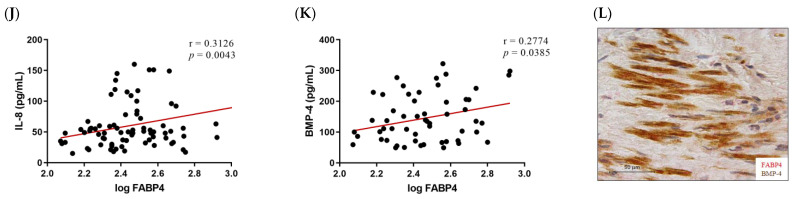
The associations of FABP4 with inflammatory, apoptotic, and calcification markers in AVs from male AS patients. Correlations between the protein levels of FABP4 and the inflammatory molecules (**A**) IL-6 and (**B**) Rantes. (**C**) The representative microphotograph of FABP4 (red)/Rantes (brown) double immunostaining in the AV sections of male AS. Correlations between the protein levels of FABP4 and the apoptotic markers (**D**) Bax, (**E**) caspase-3, and (**G**) Bcl-2. Representative microphotographs of FABP4 (red)/caspase-3(brown) (**F**) double immunostainings in AV sections of male AS. Correlations between the protein levels of FABP4 and the calcification markers (**H**) BMP-2, (**J**) IL-8, and (**K**) BMP-4. The representative microphotographs of (**I**) FABP4/BMP-2 and (**L**) FABP4/BMP-4 double immunostainings in the AV sections of male AS. AV: aortic valve; AS: aortic stenosis; FABP4: fatty acid-binding protein 4; IL: interleukin; Bcl-2: B-cell lymphoma 2; BMP: bone morphogenetic protein. N = 33–116.

Given the higher expression of FABP4 in male AVs, we investigated the possible correlations between protein levels of FABP4 and markers assessing inflammation, apoptosis, and calcification in the male AVs. FABP4 was directly correlated with the inflammatory markers IL-6 (r = 0.3389, *p* = 0.0034) and Rantes (r = 0.3023, *p* = 0.0025) (Figure 3A,B). Co-expression of FABP4 with Rantes visualized by immunohistochemistry reinforced this association (Figure 3C). The pro-apoptotic markers Bax and caspase-3 directly and significantly correlated with the FABP4 levels (r = 0.3933, *p* = 0.0075, and r = 0.4094, *p* = 0.0058, respectively) (Figure 3D,E). Moreover, a co-localization of FABP4 and caspase-3 was found in the AV sections (Figure 3F). The anti-apoptotic marker Bcl-2 was inversely correlated with FABP4 (r = −0.4096, *p* = 0.0246) (Figure 3G). Finally, the calcification markers BMP-2 (r = 0.3032, *p* = 0.0024), IL-8 (r = 0.3126, *p* = 0.0043), and BMP-4 (r = 0.2774, *p* = 0.0385) presented direct correlations with the FABP4 levels in the AVs analyzed (Figure 3H,J,K). In agreement with these results, FABP4 was co-expressed with BMP-2 and BMP-4 in male AVs (Figure 3I,L). In relation to AVs in females, we did not find any of these significant associations (data not shown).

### 2.5. FABP4 Expression Is Upregulated in Male VICs during Osteogenesis

**Figure 4:**
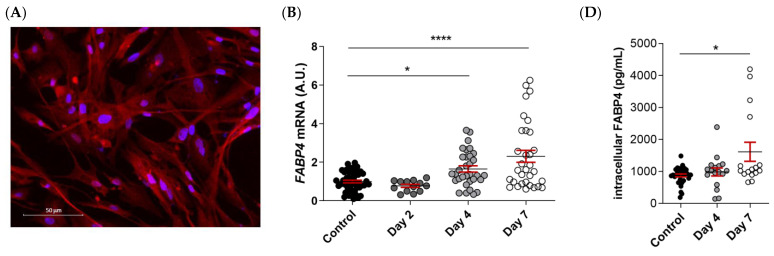

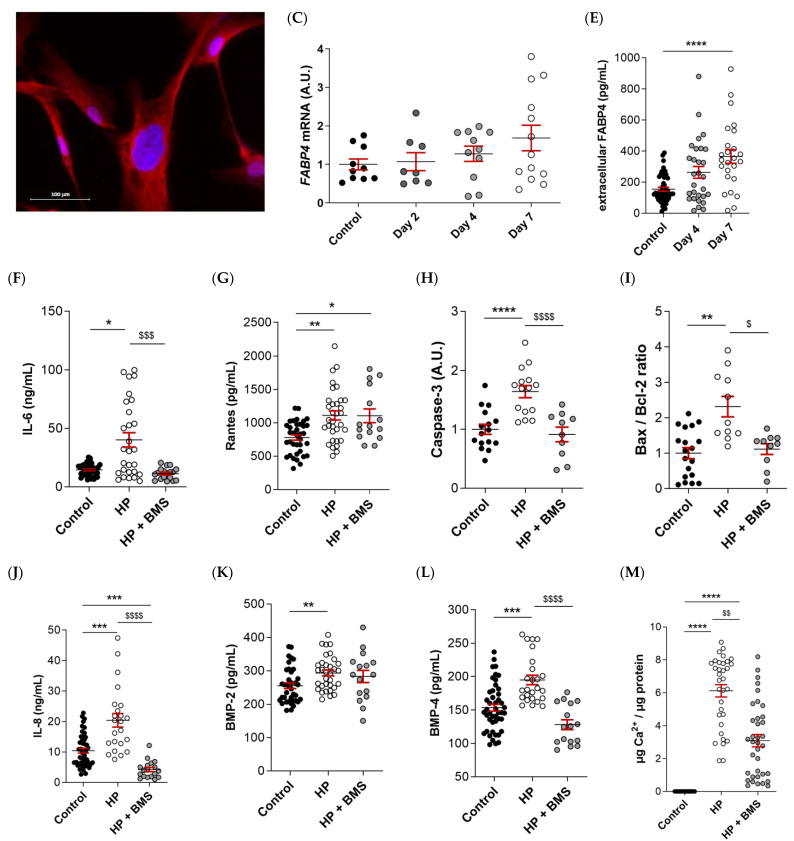
The FABP4 expression in the cultured VICs from AV of AS patients. (**A**) Representative microphotographs of immunofluorescence of FABP4 (red) counter-stained with DAPI (blue) in VICs derived from the AV of men and women. Time-course mRNA expression of *FABP4* in (**B**) male and (**C**) female VICs cultured in osteogenic media at days 2, 4, and 7. (**D**) Intracellular and (**E**) extracellular protein levels of FABP4 in male VICs cultured in osteogenic media at day 7. Protein levels of inflammatory markers (**F**) IL-6 and (**G**) Rantes and the osteogenic markers (**J**) IL-8, (**K**) BMP-2, and (**L**) BMP-4 in male VICs’ supernatant cultured in osteogenic media with or without BMS at day 7. Protein levels of pro-apoptotic marker (**H**) caspase-3 and the (**I**) Bax/Bcl-2 ratio in male VICs’ cell extract cultured in osteogenic media with or without BMS at day 7. (**M**) The calcium content in the male VICs’ cell extract cultured in osteogenic media with or without BMS at day 7. Dot plots represent the mean and standard error of the mean (SEM) of each group of subjects (men N = 3 and women N = 3, VICs from each patient have 6–9 replicates). Protein levels were measured by Western blot (normalized to stain free protein, A.U.: arbitrary units) or ELISA. The mRNA expression levels (A.U.) were normalized to the *18S*, *HPRT*, *ACTB*, and *GADPH* levels and a control group was used as the calibrator (VICs cultured in basal cell media). The calcium content was normalized to the total protein in the respective cell monolayer (µg Ca^2+^/µg protein). AV: aortic valve; AS: aortic stenosis; FABP4: fatty acid-binding protein 4; HP: High phosphate; IL: interleukin; Bcl-2: B-cell lymphoma 2; BMP: bone morphogenetic protein. Control vs. HP: * *p* < 0.05, ** *p* < 0.01, *** *p* < 0.001, **** *p* < 0.0001. Control vs. HP + BMS: $ *p* < 0.05, $$ *p* < 0.01 vs., $$$ *p* < 0.001, $$$$ *p* < 0.0001.

FABP4 was spontaneously expressed in VICs from AS patients, as evidenced by immunofluorescence (Figure 4A). Male and female VICs were cultured in osteogenic media for 2, 4, and 7 days. The mRNA expression levels of *FABP4* increased significantly at days 4 and 7 only in the male VICs (Control: 1 ± 0.07 vs. d4: 1.165 ± 0.16; *p* < 0.05, and d7: 2.3 ± 0.31; *p* < 0.0001) (Figure 4B). In female VICs, the *FABP4* mRNA was slightly but not significantly increased by osteogenic treatment (Figure 4C). Consistently, intracellular and extracellular FABP4 protein levels were determined only in male VICs at 4 and 7 days of treatment. Both the FABP4 expression and secretion increased significantly after 7 days of culture in the osteogenic medium (intracellular, Control: 877 ± 45 vs. d7: 1614 ± 297 pg/mL; *p* = 0.0235; extracellular, Control: 155 ± 13 vs. d7: 366 ± 44 pg/mL; *p* < 0.0001) (Figure 4D,E). Given that both intracellular and extracellular expression of FABP4 was consistently up-regulated only at day 7, any further experiment on calcifying VICs was conducted at the aforementioned timepoint.

### 2.6. Pharmacological Inhibition of FABP4 Reduces Inflammation, Apoptosis, and Calcification in VICs

As expected, the osteogenic medium caused an increase in the markers of inflammation (IL-6 [Control: 14.5 ± 0.8 vs. HP: 40.1 ± 6 ng/mL; *p* = 0.03] and Rantes [Control: 779 ± 40 vs. HP: 1110 ± 68 pg/mL; *p* = 0.0012], Figure 4F,G), pro-apoptosis (caspase-3 [Control: 1 ± 0.08 vs. HP: 1.64 ± 0.9; *p* < 0.0001] and Bax/Bcl-2 [Control: 1 ± 0.15 vs. HP: 2.3 ± 0.3; *p* = 0.0018] ratio, Figure 4H,I), calcification (IL-8 [Control: 10.4 ± 0.8 vs. HP: 20.4 ± 2.2 pg/mL; *p* = 0.0006], BMP-2 [Control: 255 ± 8.5 vs. HP: 293 ± 8.9; *p* = 0.0096], and BMP-4 [Control: 153 ± 5.2 vs. HP: 195 ± 7 pg/mL; *p* = 0.0002], Figure 4J–L) as well as the calcium content in the VICs (Control: 0 ± 0 vs. HP: 3.1 ± 0.4; *p* < 0.0001) (Figure 4M) relative to the control medium at 7 days. Treatment with the FABP4 specific pharmacological inhibitor BMS309403 prevented osteogenic changes in IL-6 (HP + BMS 11.3 ± 1.2 ng/mL; *p* = 0.0007), caspase-3 (HP + BMS: 0.91 ± 0.1; *p* < 0.0001), Bax/Bcl-2 (HP + BMS: 1.11 ± 0.15; *p* = 0.025), and BMP-4 (HP + BMS: 128 ± 7 pg/mL; *p* < 0.0001) expressions, reaching values similar to the basal medium (Figure 4F,H,I,L), or even lower in the case of IL-8 (Control vs. HP + BMS: 4.2 ± 0.7 pg/mL; *p* < 0.0001) (Figure 4J). The Rantes increase was milder with the inhibitor (HP + BMS: 1106 ± 103 pg/mL; *p* = 0.0345) (Figure 4G). Importantly, treatment with BMS309403 significantly reduced the calcium content of VICs in the osteogenic medium after 7 days (HP + BMS: 3.1 ± 0.4; *p* < 0.0078) (Figure 4M).

## 3. Discussion

**Figure 5:**
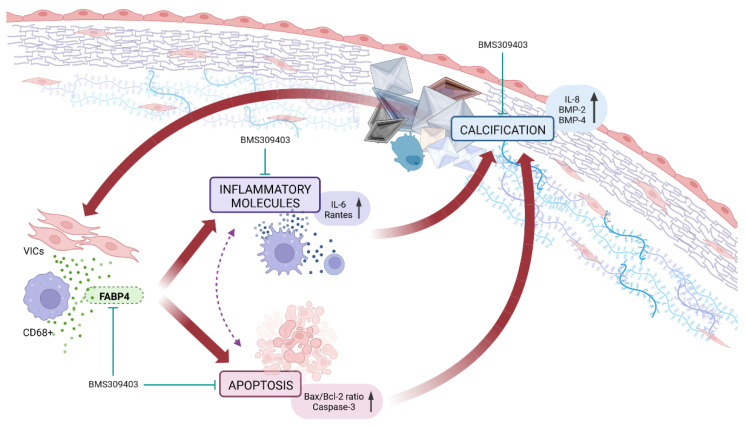
The pharmacological inhibition of FABP4 improved the VICs’ inflammation, apoptosis, and calcification in AS.

Our results demonstrate for the first time that the expression of FABP4 is enhanced in AVs from AS patients and is associated with inflammation, apoptosis, and calcification, especially in men. The VIC appears as a new source of FABP4, with a distinct sex-specific pattern. Importantly, the pharmacological blockade of FABP4 exerts beneficial effects against inflammation, apoptosis, and calcification in pro-calcifying male-derived VICs. Thus, FABP4 has emerged as a new sex-specific therapeutic target controlling inflammation, apoptosis, and calcification in AS (Figure 5).

Both adipocytes and macrophages express and secrete FABP4 [22], which is released into the bloodstream and targets several organs including the AV. We herein described that VIC also expressed FABP4, particularly in pathological conditions such as osteogenic differentiation. Moreover, FABP4 was also expressed by VECs and macrophages in the AV from AS patients. Of interest, the expression of FABP4 has been described in capillary and venous endothelial cells, but not in arterial endothelial cells in normal conditions [23], although its expression could be ectopically induced in pathological settings [24]. Importantly, FABP4 secretion occurs after raising the intracellular calcium in cells [25,26]. In agreement with this study, VICs derived from AS patients could also secrete FABP4 under pathological osteogenic stimuli, suggesting that FABP4 secretion shares common mechanisms in adipocytes, macrophages, and in VICs. The raised levels of intracellular calcium presented in the osteogenic media might lead to FABP4 secretion in male-derived VICs.

The information on the sex-differences in FABP4 expression is limited to circulating FABP4 levels. Results from the clinical studies showed an increased FABP4 plasma level in the female gender in several pathological conditions such as coronary atherosclerosis [19] or atrial fibrillation [18]. Conversely, in patients with stable angina and chronic kidney disease, male sex was associated with higher plasma FABP4 levels [20]. In our study, the FABP4 was evaluated in AVs, showing that it was overexpressed in leaflets from men compared to women. Interestingly, FABP4 was mainly expressed in the inflammatory foci (CD68+) and fibrocalcific regions, more predominant in AVs from men, as we have recently reported [9]. Interestingly, the FABP4 levels in the AVs were positively associated with inflammatory, apoptotic, and osteogenic markers in men, but not in women. The causes behind this sexual dimorphism in the tissue expression of FAPB4 are currently unknown and should be addressed in future studies. However, it is plausible to propose that there could be metabolic and/or hormonal reasons that explain these sex differences, given the association between FAPB4 levels with obesity, insulin resistance, hypertension, and atherosclerosis [12], and that different underlying cardiovascular or metabolic risk factors affect men and women differently.

Evidence for the involvement of FABP4 in inflammation and apoptosis has been accumulating. Therefore, FABP4 produced in epicardial/perivascular fat and macrophages within the atherosclerotic lesion upregulates inflammation-related pathways, leading to the development of coronary atherosclerosis [14]. FABP4 also increases inflammatory cytokines and mediates apoptosis in a variety of cell types [15,27]. The regulation of inflammation and apoptosis seems to be strongly related since FABP4 silencing protects from cardiomyocyte or chondrocyte apoptosis via the master regulator of the inflammation nuclear factor-κB (NF-κB) pathway [15,27]. Conversely, the downregulation of FABP4 using its specific inhibitor BMS309403 can suppress inflammation and/or apoptosis in pathological settings including acute lung injury, acute kidney injury, or diabetic nephropathy [27,28,29]. Of interest, treatment with BMS309403 improves insulin resistance, diabetes mellitus, fatty liver disease, and atherosclerosis [17]. In line with these publications, BMS309403 also exerted beneficial effects against inflammation and VIC apoptosis in pro-calcifying VICs. Thus, our results show that FABP4 could act in a paracrine/autocrine manner as a bioactive molecule supporting VIC inflammation and apoptosis, exerting its pharmacological inhibition beneficial effects on these pathological characteristics.

The association of FABP4 and calcification has been unexplored. Clinical studies point out that serum FABP4 is positively associated with the degree of abdominal aortic calcification in peritoneal dialysis patients [30] as well as with the presence of coronary artery calcium in patients with type-2 diabetes mellitus [31]. Our data are in agreement with these publications. First, FABP4 expression was higher in the fibrocalcific regions and positively correlated with calcification markers in AVs. Second, FABP4 expression was induced by pro-calcifying media in male VICs. Third, FABP4 pharmacological inhibition protected VICs against calcification by decreasing the osteogenic markers IL-8 and BMP-2 as well as calcium content. However, FABP4 has been identified as a key gene directly associated with calcium oxalate lithogenesis. Thus, the downregulation of FABP4 drives calcification in the development of kidney stone disease [32]. The discrepancies could be due to the differences in the pathophysiological mechanisms leading to kidney stone disease and cardiovascular diseases. In AS cohorts, dystrophic calcifications, spontaneous precipitation of calcium phosphate crystals [33], are the cause in 23% of cases [34]. However, kidney stones may account for pure calcium oxalate (50%), calcium phosphate (apatite) (5%), and a mixture of both (45%) [35].

In conclusion, FABP4 is associated with features that characterize the development and progression of AS; namely, inflammation, VIC apoptosis, and calcification of the AV. FABP4 expression exhibits a sex-specific pattern, its role being more relevant in men. Our findings support the notion that sex-specific pharmacological inhibition of FABP4 could represent a new therapeutic strategy for the management of AS patients, principally in men.

The current study has several limitations that should be acknowledged with regard to the interpretation of the findings. First, AS patients had been treated with antihypertensive drugs or statins, which have been reported to modify the FABP4 levels [36,37,38]. Nevertheless, statin intake in men was higher than women, thus ruling out any effect of these drugs on the expression. Second, circulating FABP4 has not been determined. Third, the absence of representative animal models for AS limits the impact of our findings.

## 4. Materials and Methods

### 4.1. Clinical Cohort

This prospective and observational study included 226 patients with severe AS (AV area ≤1 cm^2^ and/or transaortic mean pressure gradient >40 mmHg) referred to Hospital Universitario de Navarra for surgical AV replacement from June 2013 to June 2021. Exclusion criteria were moderate or severe concomitant valvular disease, malignant tumor, infective endocarditis, and chronic inflammatory diseases. All patients were evaluated by transthoracic echocardiography. Venous blood was drawn on admission for surgery for the measurement of routine laboratory parameters.

AVs obtained from valve replacement surgery were cut in three, using one third for VIC extraction and culture, another third for protein and RNA extraction, and the last third for histology. Forty-one AVs were classified de visu in healthy or fibrocalcific section areas, analyzing their protein and RNA expression separately. As controls, noncalcified human AVs were obtained at autopsy (n = 16) and further processed as stated above. Informed consent was obtained from each patient and the control, and the study protocol conformed to the ethical guidelines of the 1975 Declaration of Helsinki, as reflected in previous approval by the institution’s human research committee (Comité Ético de Experimentación Clínica. Gobierno de Navarra, Departamento de Salud; Ethics numbers 17/2013 and PI2019/59).

### 4.2. Histology and Immunohistochemistry Evaluation

Histological determinations in AVs were performed in 5 μm-thick sections of paraffin-embedded serial sections following the protocol of the Leica BOND-Polymer Refine Detection automatic immunostainer (Leica Biosystems, Wetzlar, Germany). All solutions were filled into the bottle-Bond Open Container (Leica, Wetzlar, Germany) and registered on a computer using the Leica Biosystem program. The immunostaining program protocol includes: fixative solution, bond wash solution, blocking with common immunohistochemistry blocker and incubated with the primary antibody for bone morphogenic protein (BMP)-2 (1/150; Abcam, Cambridge, UK), BMP-4 (1/100; Abcam, Cambridge, UK), CD45 (1/30,000; Santa Cruz Biotechnology, Dallas, TX, USA), CD68 (1/200; Santa Cruz Biotechnology, Dallas, TX, USA), FABP4 (1/100; Santa Cruz Biotechnology, Dallas, TX, USA), Rantes (1/100; Santa Cruz Biotechnology, Dallas, TX, USA), VE-Cadherin (1/150; Santa Cruz Biotechnology, Dallas, TX, USA), and Vimentin (1/1000; Santa Cruz Biotechnology, Dallas, TX, USA), followed by a post primary poly-HRP-IgG incubation. The signal was developed by using the DAB substrate. Incubation without the primary antibody was carried out in the negative controls.

### 4.3. Cell Isolation and Culture

Human aortic VICs were isolated from 20 AVs (four women and 16 men) obtained during surgical AV replacement. VICs from each patient were isolated and individually assayed, as previously described [9,10]. In brief, AVs were minced and subjected to enzymatic digestion in buffered collagenase II solution (240 U/mg of tissue, Worthington Biochemical Corporation) for 1 h, and then pelleted by centrifugation. Extracted VICs were cultured in DMEM-F12 (Gibco, Waltham, MA, USA) supplemented with 20% fetal bovine serum (FBS) (Gibco, Waltham, MA, USA), 1% penicillin/streptomycin (Gibco, Waltham, MA, USA), 50 ng/mL of insulin (Sigma Aldrich, St. Louis, MO, USA), and 10 ng/mL of FGF-2 (Novus Biological, Littleton, CO, USA) at 37 °C and 5% CO_2_ in a humidified incubator (Panasonic, Osaka, Japan). The culture media were changed every 3 days.

Experiments were performed in serum-starvation conditions (1% FBS) in multi-well plates (Sarstedt, Hildesheim, Germany) and they were carried out at passages 3–5. To induce calcification, the VICs were cultured in osteogenic medium [DMEM 1 g/L (Gibco, Waltham, MA, USA), supplemented with 1% FBS (Gibco, Waltham, MA, USA), 1% penicillin/streptomycin (Gibco, Waltham, MA, USA), 50 ng/mL of insulin (Sigma Aldrich, St. Louis, MO, USA), 10 ng/mL of fibroblast growth factor (FGF)-2 (Novus Biological, Littleton, CO, USA), and 2.6 mM phosphate buffer (Na_2_HPO_4_/NaH_2_PO_4_, pH 7.4)], switching it every day during 7 days. Cells were treated daily for 1 h with a selective FABP4 inhibitor, BMS309403 at 30 µM (Bio-Techne R&D Systems, Minneapolis, MN, USA). RNA was extracted from cells at days 2, 4, and 7. Supernatants and proteins were collected at days 4 and 7 unless otherwise indicated. At least three biological replicates (donors) per sex were used in each experiment with six technical replicates per condition.

### 4.4. Immunofluorescence

VICs were cultured on coverslips until full confluence, fixed with 4% paraformaldehyde (Sigma, St. Louis, MO, USA) for 15 min at room temperature, followed by a blockade with PBS/BSA 1% for 30 min, and permeabilization with PBS/Triton 0.2% for 30 min at RT. Primary antibody (anti-FABP4) (Santa Cruz Biotechnology, Dallas, TX, USA) was incubated for 1 h at RT. The secondary antibody (1:200 Alexa Fluor-tagged mouse secondary antibody, Thermo Fisher, Waltham, MA, USA) was incubated for 30 min at RT in the dark. Finally, the cells were mounted with DAPI-mounting media (Thermo Fisher, Waltham, MA, USA).

### 4.5. Western Blot Analysis

Commercial lysis buffer with protease inhibitors (cOmplete™ Lysis-M, Merck, Kenilworth, NJ, USA) was used for tissue and cells protein extraction. A total of 20 µg of total protein from AVs or VICs lysates were electrophoresed on commercially prepared SDS polyacrylamide gels (4–15% Criterion™ TGX Stain-Free Gel, Bio-Rad, Hercules, CA, USA), followed by a transference to the Hybond-c Extra nitrocellulose membranes (Bio-Rad, Hercules, CA, USA). Primary antibodies detecting Bax (1:100, Santa Cruz Biotechnology, Dallas, TX, USA), Bcl-2 (1:100, Santa Cruz Biotechnology, Dallas, TX, USA), caspase-3 (1:100, Santa Cruz Biotechnology, Dallas, TX, USA), FABP4 (1:50, Santa Cruz Biotechnology, Dallas, TX, USA), poly (ADP-ribose) polymerase-1 PARP-1 (1:100, Santa Cruz Biotechnology, Dallas, TX, USA), and Rantes (1:100, Santa Cruz Biotechnology, Dallas, TX, USA) were used. After washing, membranes were incubated with the HRP linked secondary antibody (1:250, Merck, Kenilworth, NJ, USA), followed by three washes (with TBS Tween-20 0.05%) and detection with an ECL Chemiluminescence Kit (Amersham, GE Healthcare, Thermo Fisher Scientific, Waltham, MA, USA). Blot densitometry analyses were performed using Image Lab software. Stain free were used as loading controls for normalization and the net band densitometry was expressed as arbitrary units (AU). Images were acquired with the Chemidoc MP Imaging system (Bio-Rad, Hercules, CA, USA). All Western blots were performed at least in triplicate for each experimental condition.

### 4.6. ELISA

BMP-2, BMP-4, FABP4, interleukin (IL)-6, IL-8 and Rantes were measured in the AV tissue homogenates and VIC supernatant following the manufacturer’s instructions (R&D Systems).

### 4.7. Quantitative Reverse Transcription PCR

The total RNA from the AVs and cells was extracted with the Trizol reagent (PRImeZOL, Canvax, Córdoba, Spain) according to the manufacturer’s instructions. Quantification and purity of the RNA samples was assessed (Epoch™ Microplate Spectrophotometer, BioTek™, Winooski, VT, USA). Only samples with A260/A280 ratio values between 1.8–2 were considered valid. First strand cDNA was synthesized with the Script™ Advanced cDNA Synthesis Kit for RT-qPCR (BioRad, Hercules, CA, USA). Quantitative PCR was performed with iQ SYBR Green Supermix (BioRad, Hercules, CA, USA) with specific primers for *FABP4* (F: 5′-TACTGGGCCAGGAATTTGAC-3′, R: 5′-TTTCCATCCCATTTCTGCAC-3′) with CFX Connect Real-Time System (BioRad) equipment. The relative quantification was calculated with MyiQ (Bio-Rad, Hercules, CA, USA) software according to the manufacturer’s instructions. Data were normalized to *18S*, *HPRT*, *ACTB*, and *GADPH* levels, and expressed as fold-change relative to healthy AS valve tissue or the control group in the VIC experiments. All PCRs were performed at least in triplicate for each experimental condition.

### 4.8. Calcium Content Determination

The calcium concentration in the VICs assayed was determined by a commercial Calcium Colorimetric Assay Kit (Sigma-Aldrich, St. Louis, MO, USA) based on the chromogenic complex formed between calcium ions and o-cresolphthalein. Calcium extraction was performed with 0.6 N HCl solution. Calcium content was normalized against the total protein content (µg Ca^2+^/µg protein).

### 4.9. Statistical Analyses

The quantitative variables are presented as the mean ± standard error of mean (SEM). Data normality was assessed by the Shapiro–Wilk and Kolmogorov–Smirnov tests. The FABP4 protein levels in AV tissue are presented as log-transformed. Differences between the two groups were analyzed by the Student *t* test if the data fitted normality, or the Mann–Whitney U test otherwise. Correlations were assessed by the Pearson correlation coefficient (r). In vitro data were analyzed using one way ANOVA (followed by the Tukey test to assess specific differences among conditions) or the Kruskal–Wallis test (with post hoc Dunn’s test for multiple comparisons between groups), depending on whether they followed a normal distribution or not, respectively. GraphPad Prism v.8.4.0 software (GraphPad Software, San Diego, CA, USA) was used for all analyses. A *p* value < 0.05 was considered statistically significant.

## Data Availability

The data presented in this study are available on request from the corresponding author.
